# The effect of dextrose prolotherapy versus placebo/other non-surgical treatments on pain in chronic plantar fasciitis: a systematic review and meta-analysis of clinical trials

**DOI:** 10.1186/s13047-023-00605-3

**Published:** 2023-02-10

**Authors:** Tannaz Ahadi, Masumeh Bagherzadeh Cham, Mahtab Mirmoghtadaei, Gholam Reza Raissi, Lobaneh Janbazi, Ghazal Zoghi

**Affiliations:** 1 Department of Physical Medicine and Rehabilitation, School of Medicine, Neuromusculoskeletal Research Center, Iran University of Medical Sciences, Tehran, Iran; 2 Endocrinology and Metabolism Research Center, Hormozgan University of Medical Sciences, Bandar Abbas, Iran

**Keywords:** Chronic plantar fasciitis, Dextrose prolotherapy, Pain, Review, Meta-analysis

## Abstract

**Background:**

Prolotherapy is the injection of a small volume of sclerosing or irritant solutions into an injured tissue. We aimed to investigate the effect of dextrose prolotherapy (DPT) versus placebo/other non-surgical treatments on pain in chronic plantar fasciitis.

**Methods:**

We searched seven electronic databases (PubMed/MEDLINE, Web of Science, EMBASE, Scopus, ProQuest, CENTRAL, PEDro) from inception to December 31, 2021 with no language restriction for publications comparing the effect of DPT with placebo/other non-surgical treatments in patients with chronic plantar fasciitis. Our primary outcome was pain and the secondary outcomes were foot function and plantar fascia thickness. The risk of bias was assessed using the Cochrane Collaboration’s tool.

**Results:**

Overall, eight studies with a total of 449 patients were included in the meta-analysis. All the included studies reported short-term pain. A large effect size (dppc2 = -0.97, 95% confidence interval [CI] -1.84 to -0.10) was observed favoring the use of DPT to reduce pain in patients with chronic plantar fasciitis in the short-term. The results for foot function improvement (dppc2 = -1.28, 95% CI -2.49 to -0.07) and plantar fascia thickness reduction (dppc2 = -1.02, 95% CI -1.99 to -0.05) in the short-term were also in favor of DPT.

**Conclusions:**

Since almost all the included studies had high risk of bias and multiple trials lacked long-term follow-ups, further high-quality research is required to determine the long-term effects of DPT vs placebo/other non-surgical interventions.

**Supplementary Information:**

The online version contains supplementary material available at 10.1186/s13047-023-00605-3.

## Introduction

Plantar fasciitis, occurring as a result of degeneration and localized inflammation of the proximal plantar aponeurosis, is among the most common causes of foot pain, affecting approximately 10% of the population in their lifetime [1, 2]. Although nonsteroidal anti-inflammatory drugs (NSAIDs) are considered the first-line medical option for the treatment of plantar fasciitis, they may not be effective for chronic cases, as their long-term use can have multiple unfavorable side effects [1, 3]. Corticosteroid and platelet-rich plasma (PRP) injections, exercise, orthoses, prolotherapy, and extracorporeal shockwave therapy (ESWT) are other treatment options for plantar fasciitis [4–6].

Prolotherapy, which is the injection of a small volume of a sclerosing or an irritant solution into an injured tissue, has gained popularity for the treatment of plantar fasciitis [7]. It is known to promote tissue regeneration and repair, release substance P, and induce fibroblast activity and vascular growth [8, 9]. Studies have shown promising results with prolotherapy for plantar fasciitis [1, 4, 10–16].

Previous systematic reviews and meta-analyses in this regard have either evaluated the effects of prolotherapy on musculoskeletal pain or lower limb tendinopathy and fasciopathy, or investigated other treatment strategies for the treatment of plantar fasciitis [17–20]. A very recent systematic review and meta-analysis evaluated the effectiveness of dextrose prolotherapy in plantar fasciitis and reported potential long-term benefits for dextrose prolotherapy [21]; however, some relevant studies were not included. Therefore, we aimed to perform a more comprehensive systematic review and meta-analysis of clinical trials evaluating the efficacy of dextrose prolotherapy versus other non-surgical treatments on pain in chronic plantar fasciitis.

## Methods

We have registered the protocol of this systematic review in the international prospective register of systematic reviews (PROSPERO) under the code CRD42020211111, available at https://www.crd.york.ac.uk/prospero/display_record.php?RecordID=211111. This protocol is in accordance with the Preferred Reporting Items for Systematic Reviews and Meta-Analyses (PRISMA) guidelines [22].

Some amendments have been made to the registered protocol which are listed below with their justifications:

• We used dppc2 instead of standardized mean difference (SMD) mentioned in our PROSPERO protocol because dppc2 also takes into account before-intervention values which can significantly influence the outcomes after the interventions.
• The Cochrane Collaboration’s tool for assessing risk of bias was used instead of PEDro’s risk of bias tool based on Armijo-Livo et al.’s study, who pointed out that many trials that have adequate quality base on the PEDro cutoff of ≥5 points, do not meet the accepted quality standards such as generation of random sequence, concealment of allocation, and blinding of study assessors defined by the Cochrane risk of bias tool. Previous studies have shown that these features can have a substantial impact on the estimates of treatment effect [23].
• We expanded our search to a larger number of databases such as PEDro because it is a physiotherapy-specific database and to perform a more comprehensive search.
• We did not search Persian-language databases separately to avoid publication bias.
• In addition, we did not limit our search to English language to avoid language bias.
• We used the Mendeley desktop software because it was available free of charge and also provided more options.
• We also used the Stata software for meta-analysis instead of RevMan for its better properties.

## Study selection criteria

### PICOS criteria for the study

#### Population

Male and female patients aged ≥ 18 years with chronic plantar fasciitis were included. Patients with Achilles tendinopathy, a history of systemic inflammatory diseases such as rheumatoid arthritis and coagulopathies, history of trauma to the foot, especially the heel, uncontrolled diabetes mellitus, and those with heel pain associated with neuropathy, crystal arthropathy, or S1 radiculopathy were excluded.

#### Intervention and comparator

Studies with more than two arms in which at least one arm received prolotherapy and another received a non-surgical treatment for plantar fasciitis were included. Prolotherapy was defined as the injection of any concentration of dextrose solution with or without any concentration of lidocaine. Placebo-controlled trials were also included. Moreover, studies using exercise or physiotherapy along with prolotherapy were included.

#### Outcomes

The primary outcome of this systematic review was pain using a visual analogue scale (VAS) or a numerical rating scale (NRS) and the secondary outcome was foot function using any available scale, including foot function index (FFI), the American Orthopedic Foot and Ankle Score (AOFAS), and foot and ankle ability measure (FAAM). The follow-up time after the final session of treatment was categorized into immediate (≤ 1 month), short-term (1–3 months), intermediate-term (3–6 months), and long-term (> 6 months).

#### Study design

This systematic review included all clinical trials that compared the effect of prolotherapy with placebo/other non-surgical treatments on plantar fasciitis. Studies that were either randomized or non-randomized, with parallel or cross-over designs, single-blind, double-blind, or open-label with concurrent control groups were included.

### Eligibility criteria

All references were imported into Mendeley Desktop software at the completion of the search and duplicated records were removed. The titles and abstracts of the primary articles that were found based on the search strategy were reviewed to determine eligibility for inclusion. Then, two reviewers (M. M., M. B.) independently assessed the full text of the potentially relevant articles. In case of disagreement between these reviewers, it was resolved by discussion to achieve consensus. A third reviewer with more experience in the field (T. A.) made the final decision when they did not reach consensus.

### Search strategy

The following databases were searched from inception to December 31, 2021 with no language restriction: PubMed/MEDLINE, Web of Science, EMBASE, Scopus, ProQuest, CENTRAL via Cochrane, and PEDro. Moreover, the National Institute of Health Clinical Trials Register (https://ClinicalTrials.gov/), the IRCTN registry (https://www.isrctn.com/), and the World Health Organization (WHO) ICTRP Search Portal (https://trialsearch.who.int/) were searched for unpublished potential studies.

Relevant search terms based on the patient (plantar fasciitis) and intervention (prolotherapy) components of the current systematic review were extracted from Emtree and Medical Subject Headings (MeSH), as well as free text words. The complete search strategies for PubMed, Web of Science, EMBASE, Scopus, ProQuest, and CENTRAL are illustrated in supplementary Table 1. Furthermore, all relevant primary studies and reviews were evaluated in terms of bibliographies for additional relevant studies. Annual meetings, ProQuest, Scopus, and Web of Science were searched for theses, conference papers, and meeting proceedings.

### Data extraction

Two independent reviewers (M. M., M. B.) performed data extraction using a pre-prepared extraction form. Upon competition of this process, one of the authors (GR. R) crosschecked the extracted data to avoid inaccuracies.

### Quality (risk of bias) assessment

The Cochrane Collaboration’s tool for assessing risk of bias was used for quality assessment [24]. The following parameters were evaluated: random sequence generation, allocation concealment, performance bias, detection bias, attrition bias, and reporting bias. Two authors (L. J., G. Z.) performed quality assessment and disagreements were resolved through discussion. A third author (T. A.) was consulted when consensus was not achieved. Studies with high risk of bias in at least one of the aforementioned areas were assumed to have an overall high risk of bias. We used the Cochrane risk of bias tool instead of PEDro tool which has been documented in our PROSPERO protocol because the Cochrane tool more strictly evaluates quality standards such as random sequence generation, allocation concealment, and blinding of study assessors compared to PEDro tool. These features have been demonstrated to have a substantial impact on the treatment effect estimates [23].

### Statistical analysis

We used the Stata software (version 14.2, StataCorp LP, College Station, TX, USA) for statistical analysis. Data were quantitatively synthesized using the random effect model and were presented in a forest plot. Heterogeneity was assessed using the Q Cochrane test and the I^2^ test [25]. Heterogeneity was then interpreted as mild (0 – 39.9), moderate (40 – 69.9), severe (70 – 89.9), and highly severe (90 – 100) [26].

Subgroup analysis was used to determine the sources of heterogeneity. Subgroup variables were the number of DPT sessions, the interval between DPT sessions, performance of DPT under ultrasound guidance, gauge of the needle used for DPT, the volume and concentration of the dextrose solution, concurrent use of anesthetics, as well as the quality of the included studies such as blinding and performance, detection, attrition, and total bias.

All studies reported short-term outcomes; therefore, we used dppc2 as the effect size [27], with interpretations based on Cohen’s criteria [28]: 0.2–0.5, small effects; 0.5–0.8, medium effects; and > 0.8, large effects. We used the Campbell Collaboration online effect size calculator available at https://campbellcollaboration.org/research-resources/effect-size-calculator.html with r = 0.3. Accordingly, we used the METAN command for three variables for analysis. To convert other types of quantitative reports into mean and standard deviation (SD) we used the method proposed by Wan et al. [29].

Since one of the studies had multiple arms comparing DPT to three other non-surgical intervention, in order to avoid the unit-of-analysis error, we divided the number of participants in the DPT group by three (13, 13, and 14) and used these sample sizes for the calculation of the effect size when different control groups were concerned [25]. Also, to avoid multiplicity, we pooled the three control arms of this study in terms of mean and standard deviation.

### Assessment of publication bias

The Egger’s weighted regression test was used for the evaluation of reporting bias [30]. Besides, the “trim-and-fill” method was performed to determine the potential influence of a publication bias on the overall results [31].

### Sensitivity analysis

The jackknife method (leave-one-out) was used for sensitivity analysis to evaluate the influence of individual studies on the overall results [32].

## Results

### Identification of studies

**Fig. 1:**
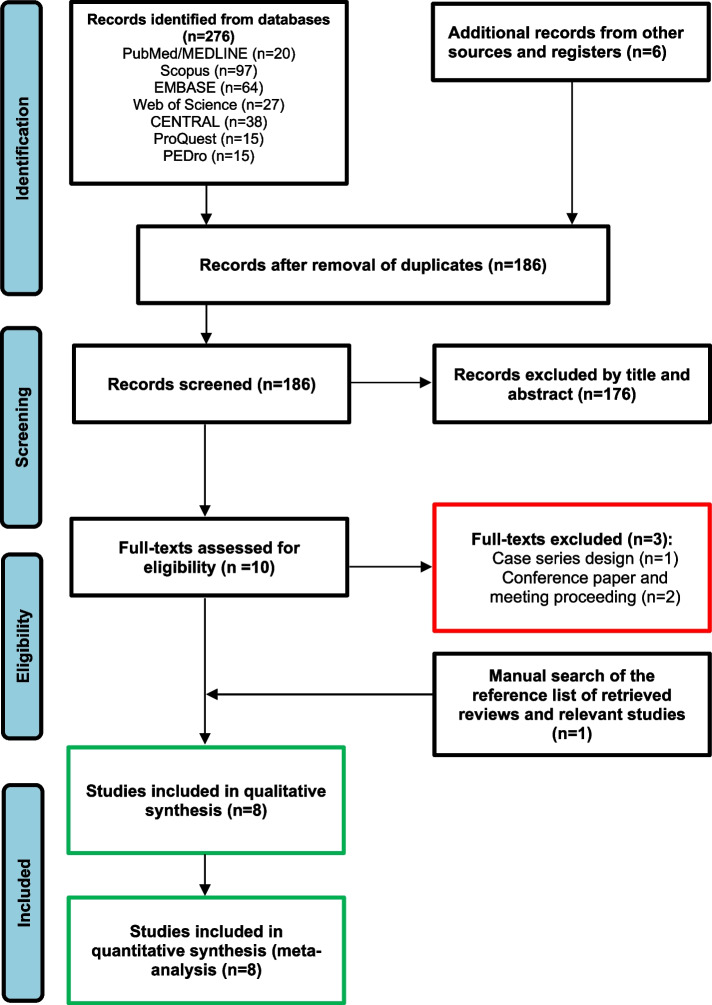
Preferred reporting items for systematic reviews and meta-analyses flow diagram

The process of study inclusion is presented in Fig. 1. A total of 276 publications were identified through searching the databases, of which 186 remained after removal of duplicates and 176 were excluded by their titles and abstracts. The full-text of the remaining publications were assessed for eligibility. Two studies were excluded because they were conference papers or meeting proceedings [33, 34] and one was excluded because it was a case-series [10]. One publication was found when the references of relevant reviews and studies were assessed. Finally, eight studies were included in the meta-analysis.

### Overview of the included studies

General characteristics of the studies are summarized in Table 1. Dextrose prolotherapy (DPT) was compared with extracorporeal shockwave therapy (ESWT) in three studies [1, 4, 16]. Platelet-rich plasma (PRP) was the comparator in two studies [15, 16]. Only one study administered DPT along with exercise, in which the control group received saline injection plus exercise [13] and in another the comparator was plantar fascia and Achilles tendon exercise [14]. Dextrose prolotherapy was compared with corticosteroid injection in two studies [11, 16].

**Table 1:** Characteristics of the included studies

Author, Year	Country	Prolotherapy group	Control group(s)	Needle	DPT interval	DPT frequency	Sample size	Age (years) Mean	Duration of symptoms Mean	Outcome measures	Follow-ups	Adverse events
Kim, 2014 [15]	Korea	Ultrasound-guided injection, 1.5 ml of 20% dextrose + 0.5 ml of 0.5% lidocaine (2 ml of 15% dextrose solution)	Ultrasound-guided injection, 2 ml of autologous PRP (Huons HC-1000 System)	22-gauge	2 weeks	2	20 DPT (*n* = 11) PRP (*n* = 9)	DPT, 37.8 PRP, 36.2	DPT, 2.9 y PRP, 2.8 y	FFI	2 weeks (before the second injection) 10 weeks (2 months after the last injection) 28 weeks (6 months after the last injection)	None
Ersen, 2018 [14]	Turkey	Ultrasound-guided injection, 3.6 ml of 15% dextrose + 0.4 ml lidocaine (4 ml of 13.5% dextrose solution)	Plantar fascia and Achilles tendon stretching exercises 3 times a week for 3 months + same exercise protocol on their own 3 times/day for the other days	27-gauge	3 weeks	3	50 DPT (*n* = 26) Exercise (*n* = 24)	DPT, 45.1 Exercise, 46.3	DPT, 32.8 m Exercise, 34.3 m	VAS, FFI, AOFAS	21 days 42 days 90 days 360 days	None
Ugurlar, 2018 [16]	Turkey	Ultrasound-guided injection, 3 ml of 5% dextrose + 1 ml of bupivacaine 5 mg/ml + 6 ml of 0.9% physiologic sodium chloride solution (10 ml of 1.5% dextrose solution)	1. ESWT, 6 Hz, 2000 pulse, 4 bars 2. PRP, 2 ml prepared by Arthrex ACP Double Syringe System 3. Corticosteroid, 1 ml betamethasone 40 mg/ml + 2 ml bupivacaine 5 mg/ml	Not specified	1 week	3	158 DPT (*n* = 40) ESWT (*n* = 39) PRP (*n* = 39) Betamethasone (*n* = 40)	DPT, 37.5 ESWT, 39.2 PRP, 38.4 Betamethasone, 40.1	DPT, 13.2 m ESWT, 15.7 m PRP, 13.9 m Betamethasone, 14.5 m	VAS, FFI-R	1 month 3 months 6 months 12 months 24 months 36 months	None
Umay Atlas, 2018 [13]	Turkey	3 ml of 15% dextrose + plantar fasciitis exercise treatment	3 ml of saline + plantar fasciitis exercise treatment	22-gauge	3 weeks	3	30 DPT (*n* = 15) Saline (*n* = 15)	DPT, 47.06 Saline, 50.60	DPT, 10.0 m Saline, 11.0 m	VAS, FFI, AOFAS	3 months (after the first injection)	None
Mansiz-Kaplan, 2020 [12]	Turkey	5 ml of 30% dextrose + 4 ml of saline + 1 ml of 2% lidocaine (10 ml of 15% dextrose)	9 ml of saline + 1 ml of 2% lidocaine	22-gauge	3 weeks	2	65 DPT (*n* = 30) Saline (*n* = 30)	DPT, 46.7 Saline, 46.2	Median of 7 m in both groups	VAS, FFI, PF thickness	7 weeks (1 month after the last injection) 15 weeks (3 months after the last injection)	None
Asheghan, 2020 [4]	Iran	Ultrasound-guided injection, 2 ml of 20% dextrose	Radial ESWT, 10 Hz, 2000 shock waves, 2 bars (3 sessions, 1 week apart)	25-gauge	1 week	2	59 DPT (*n* = 30) ESWT (*n* = 29)	DPT, 46.5 ESWT, 43.7	DPT, 4.5 m ESWT, 4.5 m	VAS, FAAM, PF thickness	6 weeks (after the first treatment session) 12 weeks (after the first treatment session)	None
Raissi, 2021 [11]	Iran	Ultrasound-guided injection, 2 ml of 20% dextrose + 1 ml of 1% lidocaine hydrochloride (3 ml of 13.33% dextrose solution)	Ultrasound-guided injection, 1 ml of 40 mg methylprednisolone plus 1 ml of normal saline + 1 ml of 1% lidocaine hydrochloride	22-gauge	-	1	40 DPT (*n* = 20) Methylprednisolone (*n* = 20)	DPT, 50.3 Methylprednisolone, 42.15	> 8 weeks	NRS, FAAM, PF thickness	2 weeks 12 weeks	None
Kesikburun, 2021 [1]	Turkey	Ultrasound-guided injection, 1.5 ml of 30% dextrose + 1.5 ml of 2% lidocaine (3 ml of 15% dextrose)	Focused ESWT 4–6 Hz, 1800–2000 shock waves + Radial ESWT, 15–21 Hz, 3000–3500 pulses, 1.8–3.0 bars (3 sessions, 2 weeks apart)	25-gauge	2 weeks	3	27 DPT (*n* = 15) ESWT (*n* = 14)	DPT, 57.4 ESWT, 51.2	DPT, 12.6 m ESWT, 12.7 m	VAS, FFI	6 weeks (after the last intervention) 12 weeks (after the intervention)	None

Abbreviations: *AOFAS* American Orthopedic Foot and Ankle Score, *DPT* dextrose prolotherapy, *ESWT* extracorporeal shock wave therapy, *FAAM* foot and ankle ability measure, *FFI* foot function index, *FFI-R* revised foot function index, *NRS* numerical rating scale, *PF* plantar fascia, *PRP* platelet-rich plasma, *SD* standard deviation, *VAS* visual analogue scale

A total of 449 adult patients (mean age, 36.2–57.4 years) were evaluated in the included studies, with sample sizes ranging from 20 to 158 and plantar fasciitis symptom duration varying from eight weeks to 2.9 years. The concentration of the dextrose solution ranged from 1.5% [16] to 20% [4]. Dextrose was combined with anesthetics such as lidocaine and bupivacaine in all studies except for one [4]. The injections were performed under ultrasound guidance in all studies but two [12, 13]. The frequency of DPT ranged from one to three injections, 1–3 weeks apart. The needles used for injections were 22-, 25-, and 27-gauge and one study did not report needle specifications [16].

The shortest follow-up time was two weeks and the longest 36 months. Pain was evaluated in different studies using VAS, NRS, or the pain component of the FFI. Meanwhile, foot function was assessed using FFI [12–15], revised FFI (FFI-R) [16], AOFAS [13, 14], and FAAM [4, 11]. Plantar fascia thickness was also evaluated in three studies [4, 11, 12]. No adverse events or complications were reported with interventions in any of the studies.

### Quality assessment

The results of the quality assessment are presented in Table 2. Of the eight studies, only one (1/8) had unclear risk of bias [13], while the rest (7/8) had high risk of bias based on Cochrane’s Collaboration tool [1, 4, 11, 12, 14–16]. All studies had low risk of reporting bias and random sequence generation [1, 4, 11–16]. The majority of studies had high risk of attrition bias [1, 4, 11, 12, 14, 15] and unclear allocation concealment [4, 11, 13, 14, 16].

**Table 2:** Risk of bias assessment by different items using the cochrane’s collaboration tool

Author, Year	Selection bias		Performance bias	Detection bias	Attrition bias	Reporting bias	Total	
	Random sequence generation	Allocation concealment					Score	Category
Kim, 2014 [15]	Low	High	Low	Low	High	Low	4	High
Ersen, 2018 [14]	Low	Unclear	High	Low	High	Low	3	High
Ugurlar, 2018 [16]	Low	Unclear	High	High	Low	Low	3	High
Umay Atlas, 2018 [13]	Low	Unclear	Low	Low	Low	Low	5	Unclear
Mansiz-Kaplan, 2020 [12]	Low	Low	Low	Low	High	Low	5	High
Asheghan, 2020 [4]	Low	Unclear	High	High	High	Low	2	High
Raissi, 2021 [11]	Low	Unclear	Low	Low	High	Low	4	High
Kesikburun, 2021 [1]	Low	Low	High	High	High	Low	3	High

### Immediate-term effects on pain

**Fig. 2:**
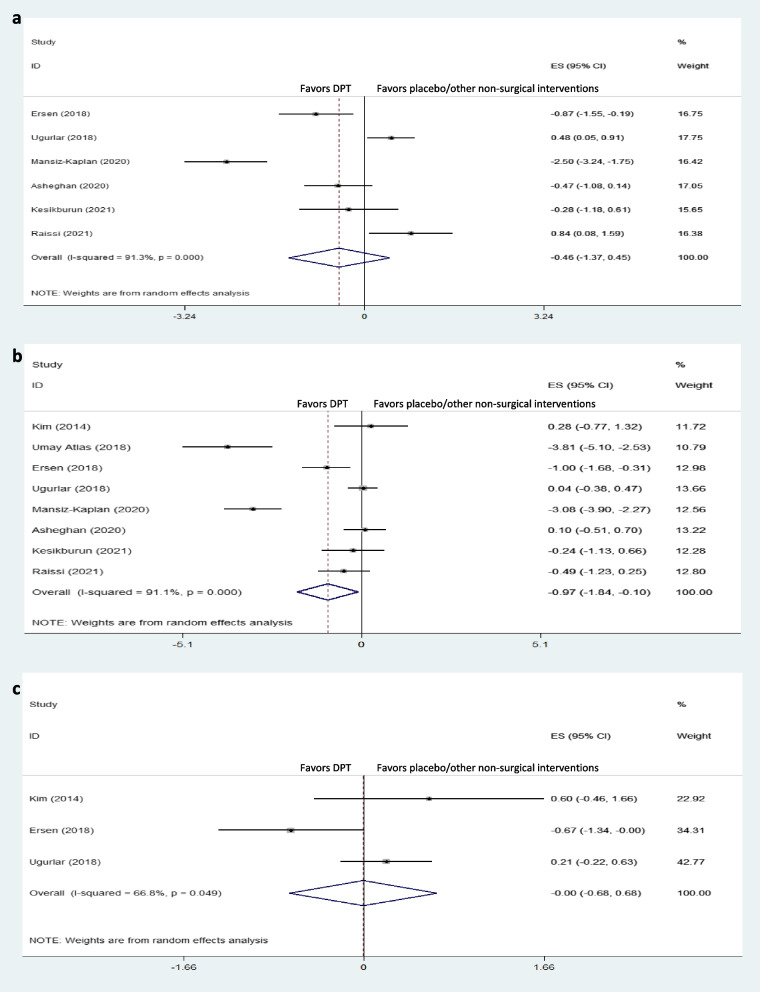
Forest plot of comparison: DPT vs placebo/other non-surgical interventions, outcome “pain”, **a**) immediate-term; **b**) short-term; and **c**) long-term

All the included trials reported the short-term effects of interventions on pain [1, 4, 11–16], while only six reported immediate-term [1, 4, 11, 12, 14, 16], and three long-term effects on pain [14–16]. Of the six studies reporting immediate-term effects on pain, Ersen et al. [14] and Mansiz-Kaplan et al. [12] showed significant immediate-term pain reduction with DPT compared to exercise and placebo, respectively. Overall, DPT was not superior to placebo/other non-surgical interventions for immediate-term pain reduction in plantar fasciitis (dppc2 = -0.46, 95% CI -1.37 to 0.45) (Fig. 2a).

### Short-term effects on pain

Pooled dppc2 showed significant large short-term effects of DPT compared to placebo/other non-surgical interventions on plantar fascia pain (dppc2 = -0.97, 95% CI -1.84 to -0.10) (Fig. 2b). Moreover, there was highly severe heterogeneity in short-term pain among the included studies (χ^2^ = 78.43, *P* = 0.00, I^2^ = 91.1%). Umay Atlas et al. showed that DPT was significantly more effective than placebo (saline) for pain reduction in the short-term [13]. Mansiz-Kaplan et al. also reported a significant short-term pain reduction with DPT compared to placebo [12]. Furthermore, Esrsen et al. illustrated similar results with DPT compared to exercise [14]. On the other hand, DPT was not superior to the control groups in this regard in other studies [1, 4, 11, 15, 16].

**Fig. 3:**
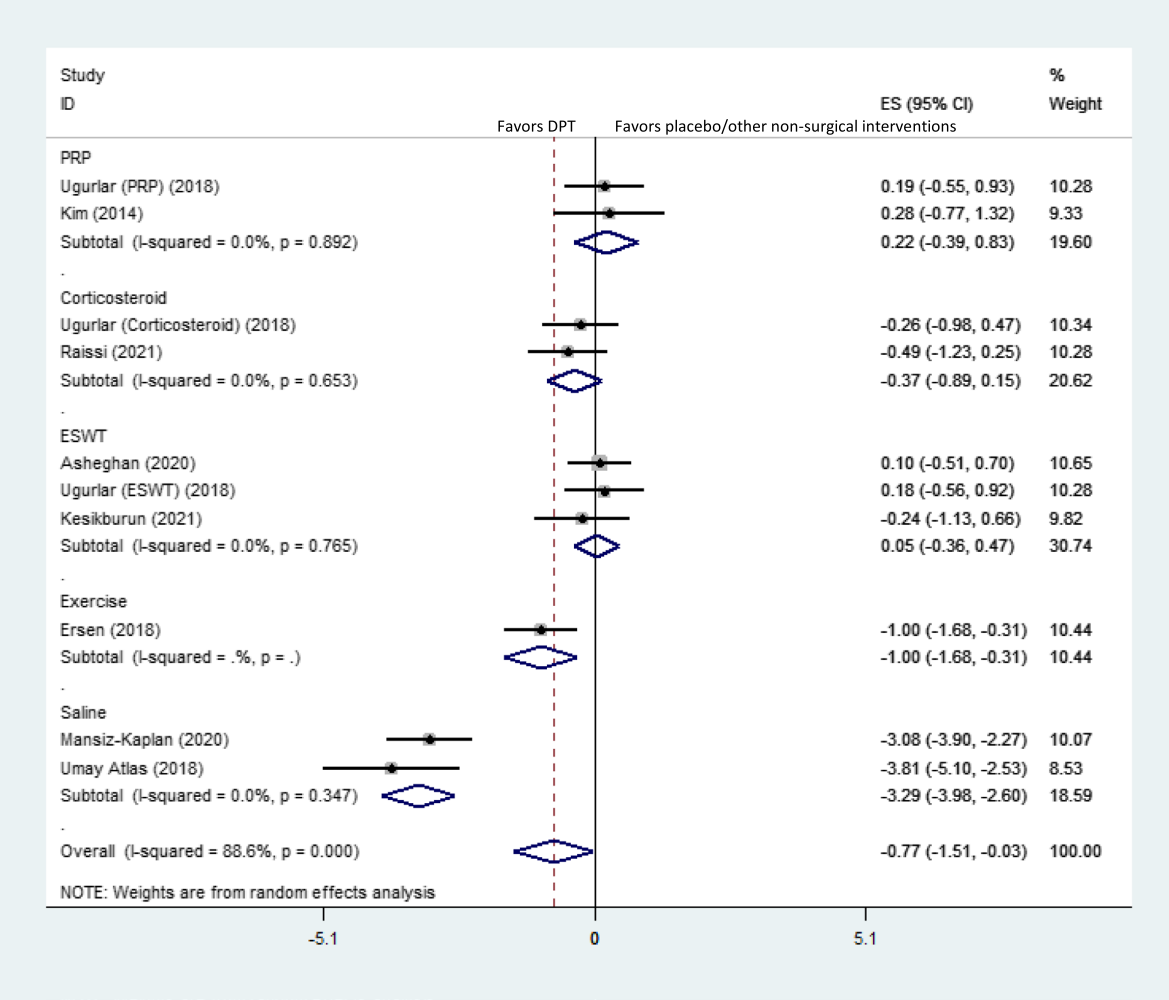
Forest plot of comparison: DPT vs placebo/other non-surgical interventions by different control groups, outcome “short-term pain”

Subgroup analysis based on different control groups showed that DPT was only significantly superior to exercise and placebo for short-term pain reduction, while it was not better than PRP, corticosteroids, or ESWT in this respect (Fig. 3).

### Long-term effects on pain

Of the three trials evaluating long-term effects on pain, only Ersen et al. reported significantly better long-term pain reduction with DPT than exercise. Also, the overall effect of DPT compared to the control groups was nonsignificant (dppc2 = 0.00, 95% CI -0.68 to 0.68) (Fig. 2c).

### Immediate-term effects on foot function

Two trials used FAAM for the evaluation of foot function [4, 11], in which an increase in the total scores indicates improvement in foot function; therefore, they were not included in the meta-analysis for foot function outcome. Of the remaining six studies, immediate-term effects on foot function was reported in 4 [1, 12, 14, 16], short-term effects in all six, and long-term effects in three [14–16].

**Fig. 4:**
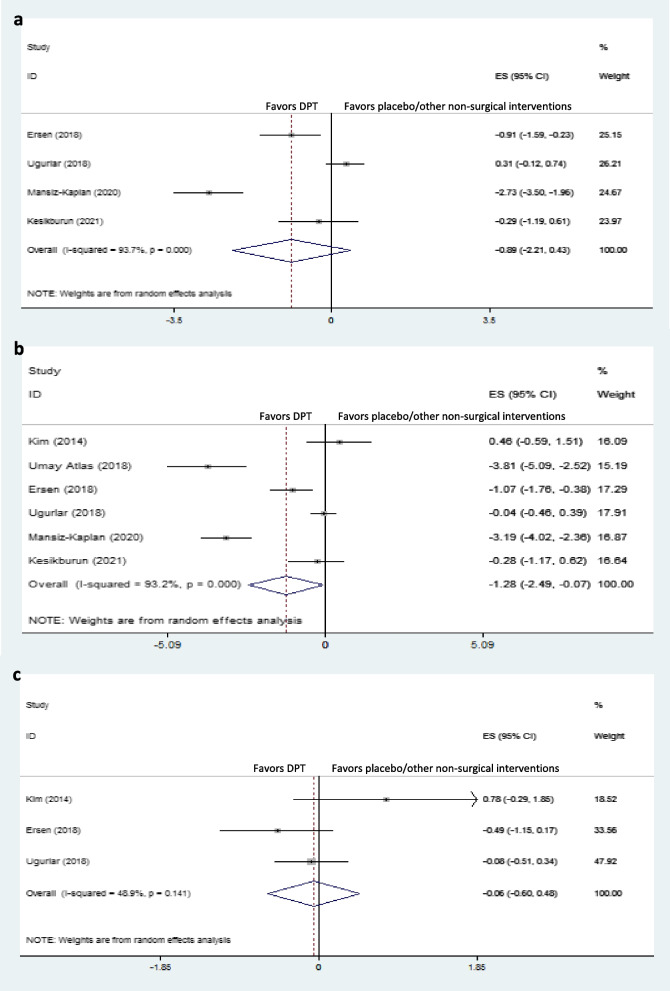
Forest plot of comparison: DPT vs placebo/other non-surgical interventions, outcome “foot function”, **a**) immediate-term; **b**) short-term; and **c**) long-term

Of the four studies reporting immediate-term effects on foot function, Ersen et al. [14] and Mansiz-Kaplan et al. [12] showed significant immediate-term foot function improvement with DPT compared to exercise and placebo, respectively. Overall, DPT was not superior to placebo/other non-surgical interventions for immediate term foot function improvement in plantar fasciitis (dppc2 = -0.89, 95% CI -2.21 to 0.43) (Fig. 4a).

### Short-term effects on foot function

Pooled dppc2 showed significant large short-term effects of DPT compared to placebo/other non-surgical interventions on foot function (dppc2 = -1.28, 95% CI -2.49 to -0.07) (Fig. 4b). Moreover, there was highly severe heterogeneity in short-term foot function among the included studies (χ^2^ = 47.50, *P* = 0.00, I^2^ = 93.2%). Umay Atlas et al. showed that DPT was significantly more effective than placebo (saline) for foot function improvement in the short-term [13]. Mansiz-Kaplan et al. also reported a significant short-term foot function improvement with DPT compared to placebo [12]. Furthermore, Esrsen et al. illustrated similar results with DPT compared to exercise [14]. On the other hand, DPT was not superior to the control groups in this respect in other studies [1, 15, 16].

**Fig. 5:**
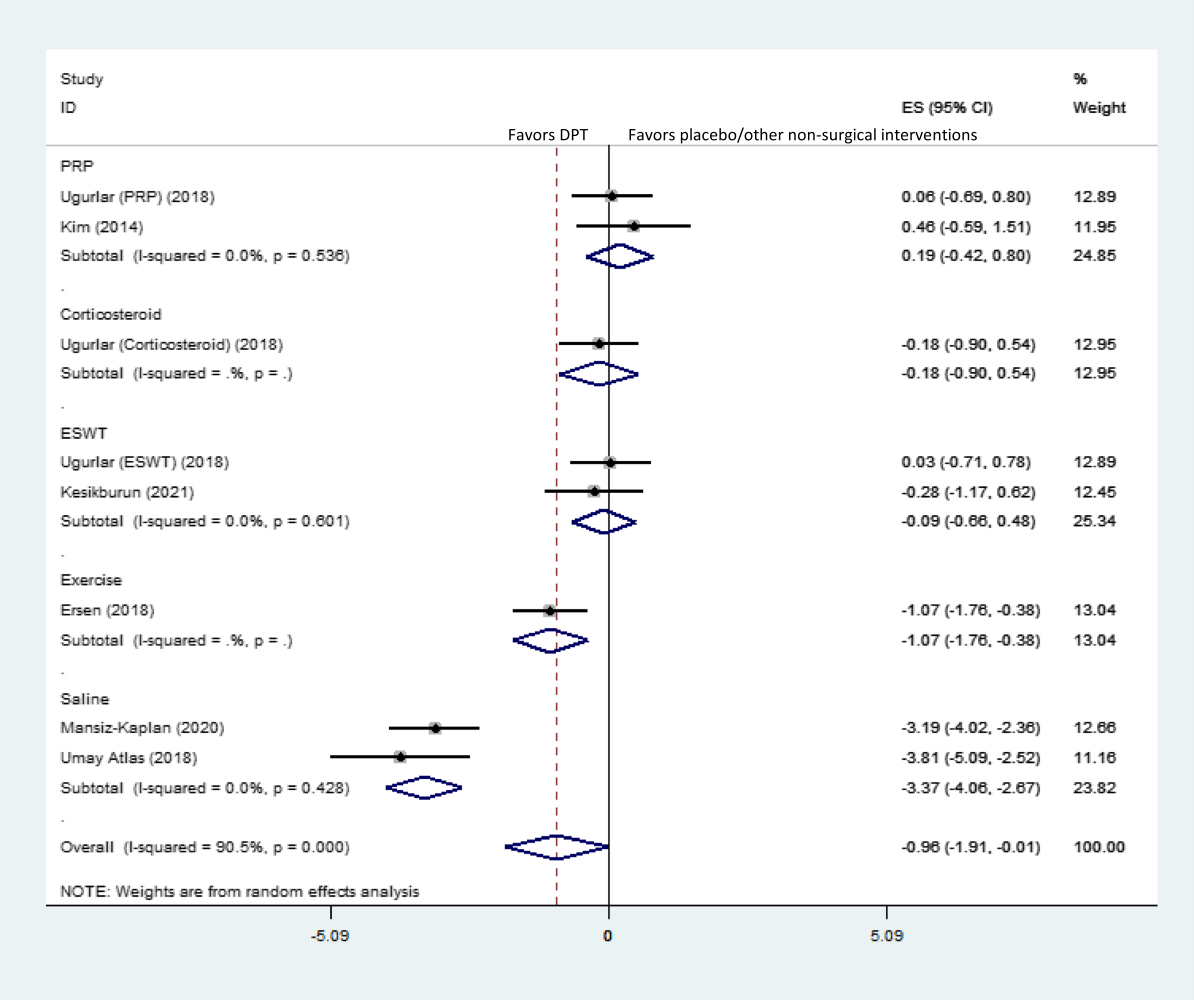
Forest plot of comparison: DPT vs placebo/other non-surgical interventions by different control groups, outcome “short-term foot function”

Subgroup analysis based on different control groups showed that DPT was only significantly superior to exercise and placebo for short-term foot function improvement, while it was not better than PRP, corticosteroids, or ESWT in this regard (Fig. 5).

### Long-term effects on foot function

Of the three trials evaluating long-term effects on foot function, none reported significantly better long-term foot function improvement with DPT than placebo/other non-surgical interventions. Also, the overall effect of DPT compared to the control groups was nonsignificant (dppc2 = -0.06, 95% CI –0.60 to 0.48) (Fig. 4c).

### Immediate-term effects on plantar fascia thickness

**Fig. 6:**
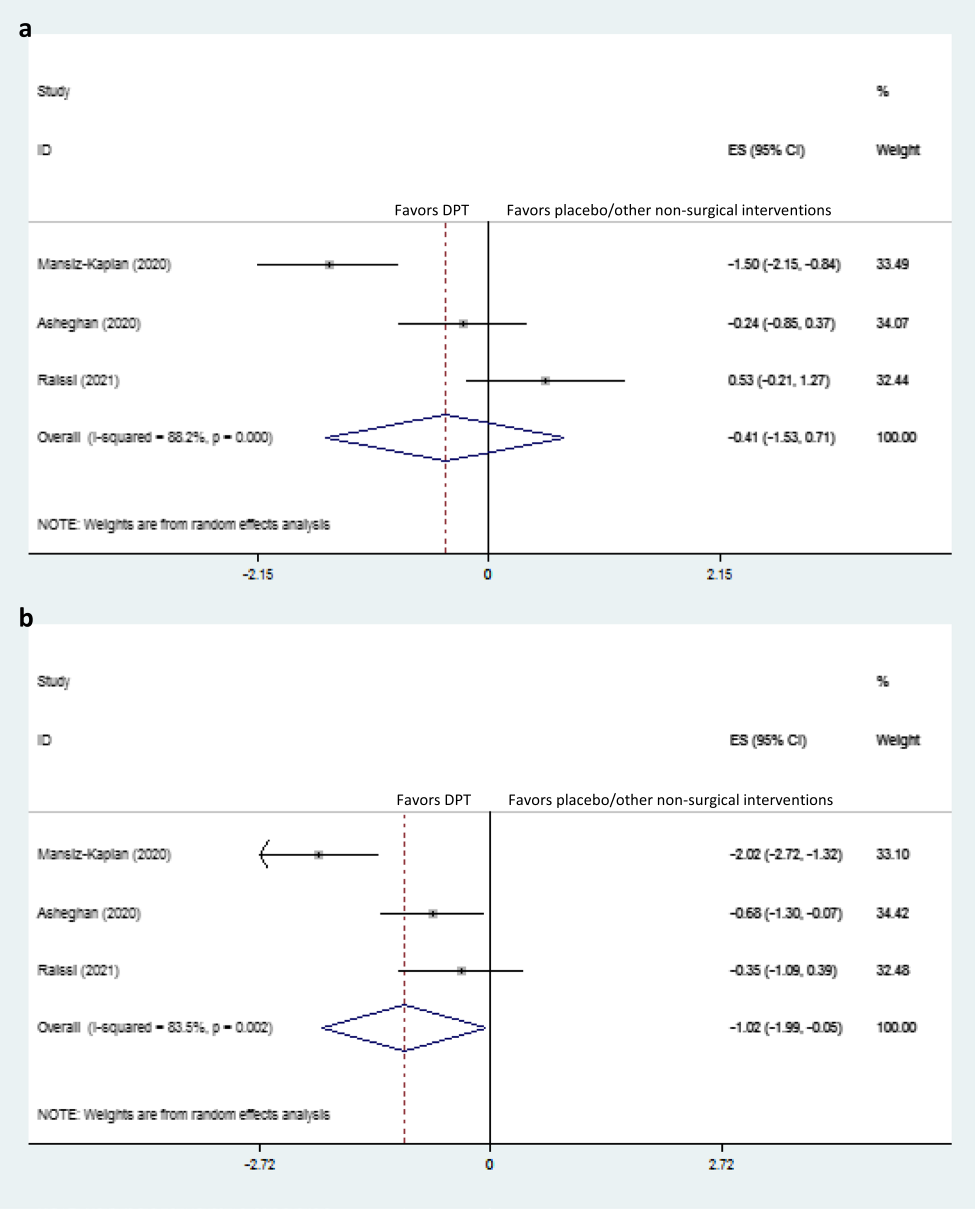
Forest plot of comparison: DPT vs placebo/other non-surgical interventions, outcome “plantar fascia thickness”, **a**) immediate-term; and **b**) short-term

Plantar fascia thickness was assessed in three studies in immediate- and short-term [4, 11, 12]. Dextrose prolotherapy significantly reduced thickness in the immediate-term compared to placebo in one study [12]. However, the overall effect of DPT compare to placebo/other non-surgical interventions on plantar fascia thickness was nonsignificant (dppc2 = -0.41, 95% CI –1.53 to 0.71) (Fig. 6a).

### Short-term effects on plantar fascia thickness

Mansiz-Kaplan et al. showed that DPT was significantly more effective than placebo (saline) for plantar fascia thickness reduction in the short-term [12]. Moreover, DPT significantly reduced plantar fascia thickness in the short-term compared to ESWT in the study by Asheghan et al. [4], while DPT was not superior to methylprednisolone injection as reported by Raissi et al. [11]. Pooled dppc2 showed significant large short-term effects of DPT compared to placebo/other non-surgical interventions on plantar fascia thickness (dppc2 = -1.02, 95% CI -1.99 to -0.05) (Fig. 6b). Also, there was severe heterogeneity in short-term plantar fascia thickness among the included studies (χ^2^ = 12.13, *P* = 0.002, I^2^ = 83.5%).

### Potential sources of heterogeneity

To determine the potential sources of the highly severe heterogeneity observed with short-term effects on pain we performed several subgroup analyses (Table 3). Accordingly, although the country in which the studies were conducted, the needle gauge used for DPT, the interval between DPT sessions, ultrasound guidance for DPT, blinding, and detection bias reduced the I^2^ index, the number of studies included in each subgroup were not sufficient to reach conclusive results. However, when studies were grouped based on having low or high performance bias, I^2^ reduced to 59.2%.

**Table 3:** Sub-group analysis for the potential heterogeneity sources of the DPT effects compared to placebo/other non-surgical interventions based on short-term pain

Potential factors		dppc2 (95% CI)	No. of studies	Heterogeneity χ^2^	*P*-value	I^2^
Country	Iran	-0.158 (-0.729, 0.413)	2	1.46	0.228	31.3%
	Turkey	-1.558 (-2.925, -0.190)	5	68.39	0.000	94.2%
	Korea	0.278 (-0.768, 1.324)	1	-	-	-
Dextrose volume	< 10 ml	-0.778 (-1.653, 0.097)	6	33.74	0.000	85.2%
	10 ml	-1.499 (-4.564, 1.566)	2	44.58	0.000	97.8%
Dextrose concentration	≥ 15%	-1.321 (-2.903, 0.261)	5	64.71	0.000	93.8%
	< 15%	-.0.437 (-1.084, 0.210)	3	6.77	0.034	70.5%
Needle	22-gauge	-1.755 (-3.570, 0.061)	4	45.10	0.000	93.3%
	25-gauge	-0.009 (-0.509, 0.492)	2	0.36	0.546	0.0%
	27-gauge	-0.997 (-1.682, -0.312)	1	-	-	-
Number of DPT sessions	1	-0.493 (-1.234, 0.248)	1	-	-	-
	2	-0.908 (-3.054, 1.238)	3	47.74	0.000	95.3%
	3	-1.138 (-2.391, 0.115)	4	34.23	0.000	91.2%
Interval between DPT sessions	None	-0.493 (-1.234, 0.248)	1	-	-	-
	1 week	0.061 (-0.286, 0.409)	2	0.02	0.892	0.0%
	2 weeks	-0.019 (-0.699, 0.661)	2	0.54	0.463	0.0%
	3 weeks	-2.577 (-4.296, -0.857)	3	22.37	0.000	91.1%
Ultrasound guidance	Yes	-0.217 (-0.589, 0.154)	6	8.78	0.118	43.1%
	No	-3.292 (-3.980, -2.605)	2	0.88	0.347	0.0%
Use of anesthetics	Yes	-0.750 (-1.671, 0.171)	6	49.26	0.000	89.8%
	No	-1.816 (-5.644, 2.013)	2	29.15	0.000	96.6%
Blinding	Yes	-1.588 (-2.921, -0.255)	5	46.96	0.000	91.5%
	No	0.022 (-0.301, 0.346)	3	0.39	0.823	0.0%
Performance bias	Low	-1.755 (-3.570, 0.061)	4	45.10	0.000	93.3%
	High	-0.236 (-0.726, 0.253)	4	7.35	0.062	59.2%
Detection bias	Low	-1.588 (-2.921, -2.255)	5	46.96	0.000	91.5%
	High	0.022 (-0.301, 0.346)	3	0.39	0.823	0.0%
Attrition bias	Low	-0.747 (-1.694, 0.201)	6	44.97	0.000	88.9%
	High	-1.834 (-5.613, 1.944)	2	31.27	0.000	96.8%
Total bias	High	-0.623 (-1.405, 0.158)	7	53.08	0.000	88.7%
	Unclear	-3.812 (-5.096, -2.529)	1	-	-	-

### Assessment of publication bias

**Fig. 7:**
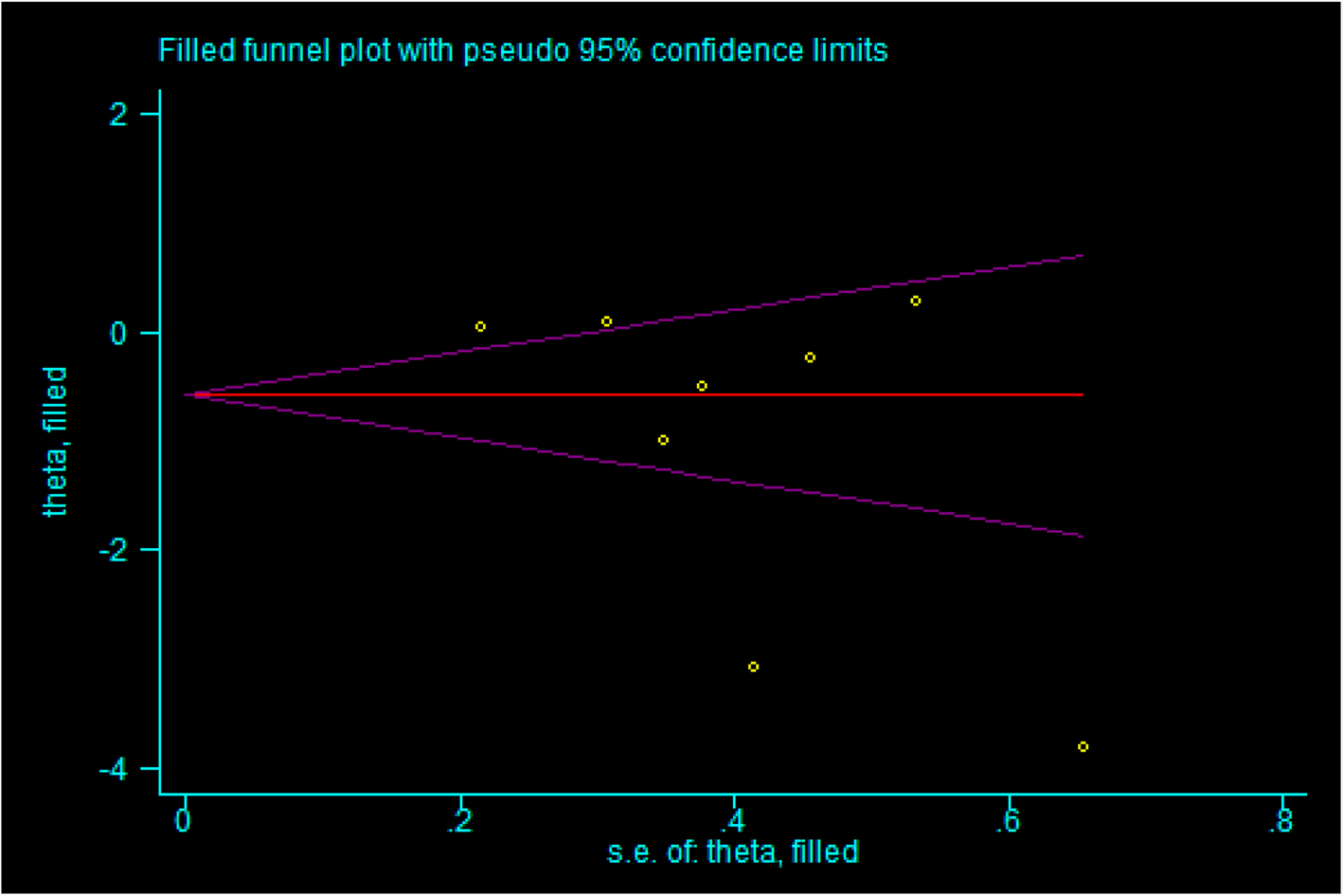
The trim-and-fill diagram for the assessment of publication bias for the outcome “short-term pain”

The results of the Egger’s linear regression (intercept = -5.66, standard error = 3.29, 95% CI -13.72 to 2.39, *P* = 0.136) showed a minor probability of publication bias. Also, the trim-and-fill method identified no missing studies leaving the pooled estimates unchanged (Fig. 7). Due to the limited overall number of trials included (< 10), we did not obtain a funnel plot.

### Sensitivity analysis

**Fig. 8:**
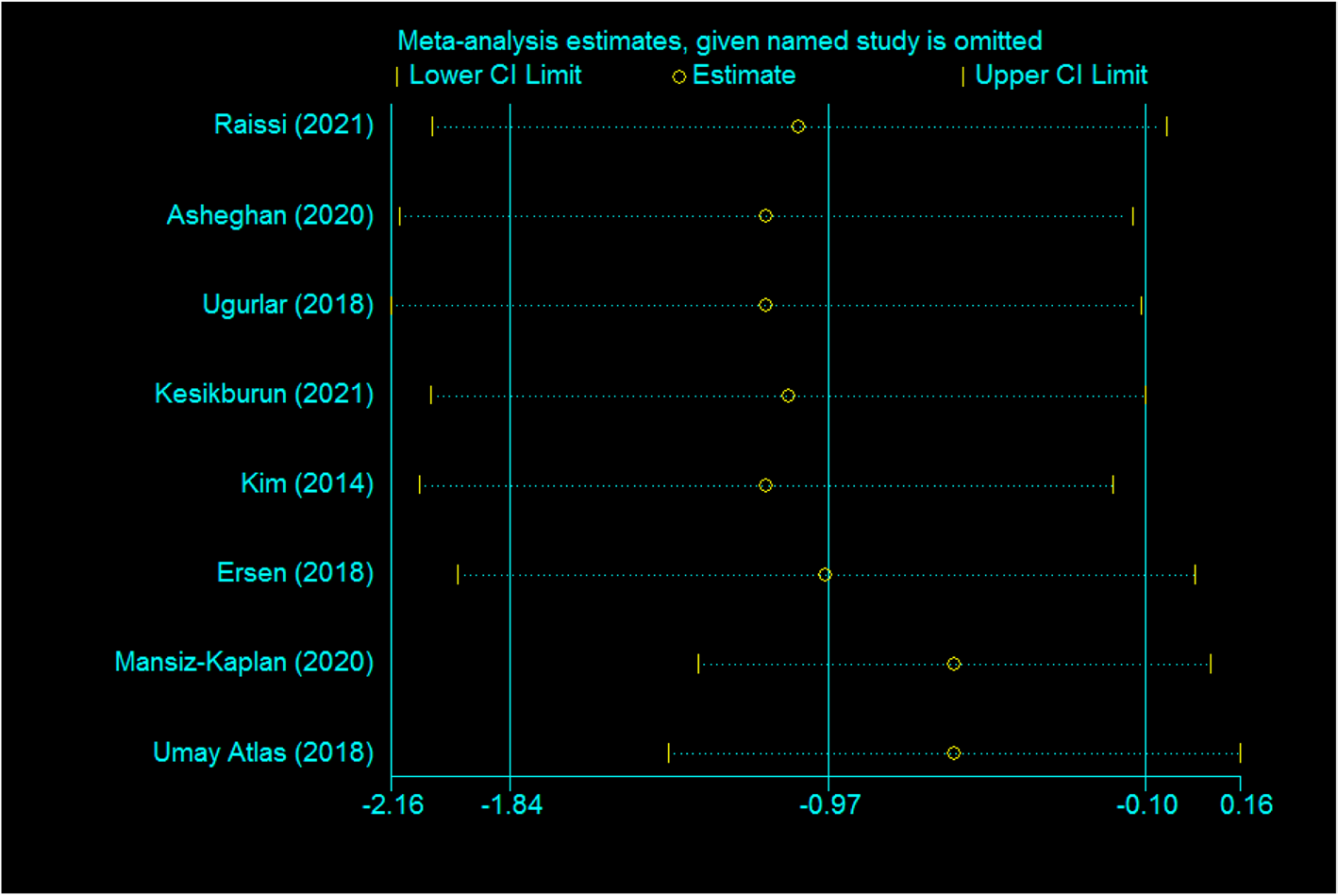
The leave-one-out sensitivity analysis for the outcome “short-term pain”

By using the leave-one-out sensitivity analysis we tried to assess the influence of individual studies on the overall estimates of the effect of DPT compared to placebo/other non-surgical interventions on short-term pain (Fig. 8). The results revealed that the included studies did not influence the pooled dppc2 of this outcome.

## Discussion

The current study systematically reviewed and meta-analyzed the efficacy of DPT on chronic plantar fasciitis compared to placebo/other non-surgical interventions. We found that overall, DPT was superior in terms of pain reduction, foot function improvement, and decreasing plantar fascia thickness in the short-term. Another finding of this review was the highly severe heterogeneity among the included studies for the short-term pain and foot function outcomes. Performance bias, i.e. the blinding of participants, was the most probable source of heterogeneity, yet it appears that this factor has not influenced the overall meta-analysis estimates.

As a non-surgical regenerative injection technique, prolotherapy is in fact the administration of an irritant solution in small amounts to a degenerated tissue [35, 36]. Although the mechanism of action of prolotherapy is not yet completely understood, growth factor stimulation through the inflammatory healing process is believed to be involved [36]. This process appears to be activated as a result of the localized trauma caused by the injection of hypertonic dextrose, leading to soft tissue healing [37–39]. In a pilot study on 20 patients with plantar fasciitis, ultrasound-guided dextrose injection led to a significant decrease in pain both at rest and during activities [10]. Another small before-and-after study on recreational athletes with chronic plantar fasciitis reported significant functional and symptomatic improvements with 15% dextrose solution [40]. The proliferative effects of DPT on the connective tissue has also been demonstrated in animal models [41–43], as well as human knee osteoarthritis and low back pain [44, 45].

In an earlier systematic review conducted by Sanderson et al. limited evidence was found regarding the safety and efficacy of DPT for lower limb tendinopathy and fasciopathy, including plantar fasciitis, Achilles tendinopathy, and Osgood-Schlatter disease [17]. In another systematic review by Hauser et al., DPT was reported to be useful for the treatment of chronic musculoskeletal pain, such as in the knee and finger joints, pelvic or spinal pain, and tendinopathies; nevertheless, they were unable to determine its effectiveness in myofascial pain [18]. A similar, more recent systematic review and meta-analysis showed the superiority of DPT to saline injection or exercise and its comparability to PRP or steroid injection in the treatment of chronic musculoskeletal pain [46]. In addition, Tsikopoulos et al. showed comparable effects for DPT and PRP in the treatment of plantar fasciitis [20]. Another systematic review and meta-analysis illustrated insufficient evidence of the clinical benefits of DPT for tendinopathies, fasciopathies and ligament injuries [47]. The contradictory findings of these systematic reviews and meta-analyses may be due to the inclusion of a broad range of conditions.

Our findings are to some extent in line with those of Lai et al. [21], who included a fewer number of trials in their meta-analysis. However, we were able to pool the eight included studies for the short-term pain and foot function outcomes, leaning towards more conclusive results. On the other hand, Lai et al. reported that DPT was inferior to corticosteroid injection in the short-term [21], which is contrary to our results. This can be justified by the different classifications of follow-up periods in their study and ours, as well as combining two heterogeneous foot function indices by Lai et al. [21].

The major strength of the current study was that by using dppc2 as the effect size we were able to perform a pooled analysis. We performed publication bias assessment and found it highly improbable. Furthermore, the sensitivity analysis using the leave-one-out method revealed that the individual included studies did not influence the pooled dppc2 of short-term pain.

Our study had several limitations. First, due to the relatively few number of the included studies, there were insufficient trials in most of the subgroups to achieve conclusive results. Second, two trials assessed the effects of DPT on foot function using the FAAM score, in which contrary to FFI and FFI-R, an increase in the total score indicated improvement in foot function; thus, their reports could not be included in the meta-analysis of the foot function outcome. Third, one trial had multiple arms and to assess each intervention against DPT we had to divide the sample size of the DPT group to avoid a unit-of-analysis error while this makes the estimates prone to multiplicity.

## Conclusions

Dextrose prolotherapy appears to be efficacious for the treatment of chronic plantar fasciitis, especially in terms of short-term pain, foot function, and plantar fascia thickness. Dextrose prolotherapy was only significantly superior to exercise and placebo for short-term pain reduction, while it was not better than PRP, corticosteroids, or ESWT in this respect. This was also the case for short-term foot function. Regarding short-term plantar fascia thickness reduction, DPT was only superior to ESWT and placebo. As performance bias was the most potential source of heterogeneity in this study, future clinical trials should consider blinding the patients where possible. Also, randomized clinical trials with lower risk of bias and longer and more frequent follow-ups are required to determine the long-term efficacy of DPT in the treatment of chronic plantar fasciitis.

## Supplementary Information

**Additional file 1.**

## Data Availability

The datasets used and/or analyzed during the current study are available from the corresponding author on reasonable request.
