# Supplementary material for: The effect of dextrose prolotherapy versus placebo/other non-surgical treatments on pain in chronic plantar fasciitis: a systematic review and meta-analysis of clinical trials
Source: J Foot Ankle Res. 2023 Feb 10;16:5. doi: 10.1186/s13047-023-00605-3 (PMC9912486; doi:10.1186/s13047-023-00605-3)
Supplement: Supplementary file 1 — Additional file 1. [file 13047_2023_605_MOESM1_ESM.docx]

Supplementary Table 1: Complete search strategies for 6 databases

| Database | Search strategy |
| --- | --- |
| Pubmed/MEDLINE | (“plantar fasci*”[all] OR “policeman’s heel*”[all] OR “policeman heel*”[all] OR (heel*[all] AND policeman*[all]) OR “policemans heel”[all] OR “heel spur syndrome”[all] OR “heel-spur syndrome”[all] OR “chronic plantar fasci*”[all] OR (chronic[all] AND “plantar fasci*”[all]) OR (fasci*[all] AND plantar[all] AND chronic[all]) OR “recalcitrant plantar fasci*”[all] OR (recalcitrant[all] AND “plantar fasci*”[all]) OR (recalcitrant[all] AND “plantar fasci*”[all]) OR “heel pain”[all] OR (chronic[all] AND “heel pain”[all]) OR “calcaneal spur syndrome”[all])  AND  (“prolotherap*”[all] OR “proliferation therap*”[all] OR (therap*[all] AND proliferation[all]) OR “dextrose prolotherap*”[all] OR (dextrose[all] AND (prolotherap*[all] OR “proliferation therap*”[all])) OR “glucose prolotherapy*”[all] OR (glucose[all] AND (prolotherap*[all] OR “proliferation therap*”[all])) OR “regenerative injection therap*”[all])  AND  (1000/01/01:2021/12/31[dp]) |
| Scopus | (TITLE-ABS("plantar fasci*") OR TITLE-ABS("policeman's heel*") OR TITLE-ABS("policeman heel*") OR (TITLE-ABS(heel*) AND TITLE-ABS(policeman*)) OR TITLE-ABS("policemans heel") OR TITLE-ABS("heel spur syndrome") OR TITLE-ABS("heel-spur syndrome") OR TITLE-ABS("chronic plantar fasci*") OR (TITLE-ABS(chronic) AND TITLE-ABS("plantar fasci*")) OR (ALL(fasci*) AND ALL(plantar) AND ALL(chronic)) OR TITLE-ABS("recalcitrant plantar fasci*") OR (TITLE-ABS(recalcitrant) AND TITLE-ABS("plantar fasci*")) OR (TITLE-ABS(recalcitrant) AND TITLE-ABS("plantar fasci*")) OR TITLE-ABS("heel pain") OR (TITLE-ABS(chronic) AND TITLE-ABS("heel pain")) OR TITLE-ABS("calcaneal spur syndrome"))  AND  (TITLE-ABS("prolotherap*") OR TITLE-ABS("proliferation therap*") OR (TITLE-ABS(therap*) AND TITLE-ABS(proliferation)) OR TITLE-ABS("dextrose prolotherap*") OR (TITLE-ABS(dextrose) AND (TITLE-ABS(prolotherap*) OR TITLE-ABS("proliferation therap*"))) OR TITLE-ABS("glucose prolotherapy*") OR (TITLE-ABS(glucose) AND (TITLE-ABS(prolotherap*) OR TITLE-ABS("proliferation therap*"))) OR TITLE-ABS("regenerative injection therap*"))  AND  PUBYEAR < 2022 |
| Web of Science | (ALL=(“plantar fasci*”) OR ALL=(“policeman’s heel*”) OR ALL=(“policeman heel*”) OR (ALL=(heel*) AND ALL=(policeman*)) OR ALL=(“policemans heel”) OR ALL=(“heel spur syndrome”) OR ALL=(“heel-spur syndrome”) OR ALL=(“chronic plantar fasci*”) OR (ALL=(chronic) AND ALL=(“plantar fasci*”)) OR (ALL=(fasci*) AND ALL=(plantar) AND ALL=(chronic)) OR ALL=(“recalcitrant plantar fasci*”) OR (ALL=(recalcitrant) AND ALL=(“plantar fasci*”)) OR (ALL=(recalcitrant) AND ALL=(“plantar fasci*”)) OR ALL=(“heel pain”) OR (ALL=(chronic) AND ALL=(“heel pain”)) OR ALL=(“calcaneal spur syndrome”))  AND  (ALL=(“prolotherap*”) OR ALL=(“proliferation therap*”) OR (ALL=(therap*) AND ALL=(proliferation)) OR ALL=(“dextrose prolotherap*”) OR (ALL=(dextrose) AND (ALL=(prolotherap*) OR ALL=(“proliferation therap*”))) OR ALL=(“glucose prolotherapy*”) OR (ALL=(glucose) AND (ALL=(prolotherap*) OR ALL=(“proliferation therap*”))) OR ALL=(“regenerative injection therap*”))  AND  PY=(1000-2021) |
| EMBASE | (‘plantar fasci*’ OR ‘policeman heel*’ OR ‘policeman heel*’ OR (heel* AND policeman*) OR ‘policemans heel’ OR ‘heel spur syndrome’ OR ‘heel-spur syndrome’ OR ‘chronic plantar fasci*’ OR (chronic AND ‘plantar fasci*’) OR (fasci* AND plantar AND chronic) OR ‘recalcitrant plantar fasci*’ OR (recalcitrant AND ‘plantar fasci*’) OR (recalcitrant AND ‘plantar fasci*’) OR ‘heel pain’ OR (chronic AND ‘heel pain’) OR ‘calcaneal spur syndrome’)  AND  (‘prolotherap*’ OR ‘proliferation therap*’ OR (therap* AND proliferation) OR ‘dextrose prolotherap*’ OR (dextrose AND (prolotherap* OR ‘proliferation therap*’)) OR ‘glucose prolotherapy*’ OR (glucose AND (prolotherap* OR ‘proliferation therap*’)) OR ‘regenerative injection therap*’)  AND  [2000-2021]/PY |
| CENTRAL | (‘plantar fasci*’ OR ‘policeman heel*’ OR ‘policeman heel*’ OR (heel* AND policeman*) OR ‘policemans heel’ OR ‘heel spur syndrome’ OR ‘heel-spur syndrome’ OR ‘chronic plantar fasci*’ OR (chronic AND ‘plantar fasci*’) OR (fasci* AND plantar AND chronic) OR ‘recalcitrant plantar fasci*’ OR (recalcitrant AND ‘plantar fasci*’) OR (recalcitrant AND ‘plantar fasci*’) OR ‘heel pain’ OR (chronic AND ‘heel pain’) OR ‘calcaneal spur syndrome’)  AND  (‘prolotherap*’ OR ‘proliferation therap*’ OR (therap* AND proliferation) OR ‘dextrose prolotherap*’ OR (dextrose AND (prolotherap* OR ‘proliferation therap*’)) OR ‘glucose prolotherapy*’ OR (glucose AND (prolotherap* OR ‘proliferation therap*’)) OR ‘regenerative injection therap*’) |
| ProQuest | (AB,TI(("plantar fascia" OR "plantar fasciitis")) OR AB,TI("policeman's heel*") OR AB,TI("policeman heel*") OR (AB,TI(heel*) AND AB,TI(policeman*)) OR AB,TI("policemans heel") OR AB,TI("heel spur syndrome") OR AB,TI("heel-spur syndrome") OR AB,TI("chronic plantar fasci*") OR (AB,TI(chronic) AND AB,TI(("plantar fascia" OR "plantar fasciitis"))) OR (ALL,FT(fasci*) AND ALL,FT(plantar) AND ALL,FT(chronic)) OR AB,TI("recalcitrant plantar fasci*") OR (AB,TI(recalcitrant) AND AB,TI(("plantar fascia" OR "plantar fasciitis"))) OR (AB,TI(recalcitrant) AND AB,TI(("plantar fascia" OR "plantar fasciitis"))) OR AB,TI("heel pain") OR (AB,TI(chronic) AND AB,TI("heel pain")) OR AB,TI("calcaneal spur syndrome"))  AND  (AB,TI("prolotherap*") OR AB,TI("proliferation therap*") OR (AB,TI(therap*) AND AB,TI(proliferation)) OR AB,TI("dextrose prolotherap*") OR (AB,TI(dextrose) AND (AB,TI(prolotherap*) OR AB,TI("proliferation therap*"))) OR AB,TI("glucose prolotherapy*") OR (AB,TI(glucose) AND (AB,TI(prolotherap*) OR AB,TI("proliferation therap*"))) OR AB,TI("regenerative injection therap*"))  AND  PY(10000101-20211231) |
